# Epidemic of wild-origin H1NX avian influenza viruses in Anhui, China

**DOI:** 10.1186/s40249-017-0304-4

**Published:** 2017-07-03

**Authors:** Ye Ge, Qiu-Cheng Yao, Xian-Fu Wang, Zhi-Qiang Fan, Guo-Hua Deng, Hong-Liang Chai, Hua-Lan Chen, Yu-Ping Hua

**Affiliations:** 1 College of Wildlife Resources, Northeast Forestry University, Harbin, 150040 Heilongjiang Province China; 2 State Key Laboratory of Veterinary Biotechnology, Harbin Veterinary Research Institute, Chinese Academy of Agricultural Sciences, Harbin, China; 3 Natural Protection & Management Station of Forestry Department Centre of Anhui Province, Hefei, Anhui Province China; 4 School of Life Sciences, Anqing Normal University, Anqing, Anhui Province China

**Keywords:** H1N1, Anseriformes, Epidemic, Phylogenetic, Pathogenic

## Abstract

**Background:**

As the natural hosts of avian influenza viruses (AIVs), aquatic and migratory birds provide a gene pool for genetic transfer among species and across species, forming transient “genome constellations.” This work describes the phylogenetic dynamics of H1NX based on the complete molecular characterization of eight genes of viruses that were collected from 2014 to 2015 in Anhui Province, China.

**Methods:**

Hemagglutination and hemagglutination inhibition tests were used to determine the hemagglutination (HA) activity of the HA subtypes. The entire genomes of the viruses were sequenced on an ABI PRISM 3500xl DNA Analyzer. The sequences were genetically analysed to study their genetic evolution using DNASTAR and MEGA 6. The pathogenic effects of the viruses were evaluated using mouse infection models.

**Results:**

Seven strains of the H1 subtype avian influenza virus were isolated. Phylogenetic analysis indicated natural recombination of the H1 influenza viruses between the Eurasian lineage and the North American lineage. Some genes had high sequence identity with A/bean goose/Korea/220/2011(H9N2), which is a typical case involving viral reassortment between the Eurasian lineage and the North American lineage. The results of infection experiments in mice showed that the viruses could acquire the ability to multiply in mouse respiratory organs without adaptation.

**Conclusions:**

These findings suggest that continued surveillance of wild birds, particularly migratory birds, is important to provide early warning of possible H1 influenza epidemics and to understand the ecology of the virus.

**Electronic supplementary material:**

The online version of this article (doi:10.1186/s40249-017-0304-4) contains supplementary material, which is available to authorized users.

## Multilingual abstracts

Please see Additional file 1 for translations of the abstract into the five official working languages of the United Nations.

## Background

H1 avian influenza viruses (AIVs) represent a virus type that can cross the species barrier from birds to mammals; these viruses can co-circulate in wild birds, pigs, and human beings [1]. Many researchers have suggested that the H1 influenza virus can be transmitted across hosts from birds to mammals and that it poses a threat to public health. Similarly, reverse transmission of the influenza virus from mammals to birds has also been reported [2, 3]. Three human epidemics have been attributed to H1N1, and these epidemics were accompanied by significant loss of life. The genes of the three H1N1 influenza viruses that caused epidemics in humans were derived in part or completely from avian viruses. As a major natural gene pool reservoir, wild birds are gaining increasing attention for their role in the transmission and reassortment of AIVs, and wild birds subsequently spread viral genes to domestic poultry, wild mammals, swine, and humans [4–8]. However, although H1-subtype influenza viruses are common pandemic strains in humans and swine, they are not common among the 16 AIV subtypes found in poultry, and they are particularly rare in wild birds.

In this context, we isolated 7 H1 AIV strains based on the surveillance of wild birds in the wetlands of the National Nature Reserves in Anhui Province in China and analysed and evaluated the evolution, biological properties, and potential threat to mammals of these novel H1NX viruses.

## Methods

### Virus isolation and identification

In 2014–2015, faecal samples, throat swabs, and cloacal swabs were collected from migrating birds and captive birds in Anhui Province, China. The samples were placed in a protective medium consisting of phosphate-buffered solution (pH 7.2) containing penicillin, streptomycin, cephalosporin, and 10% glycerin. After the samples were vortexed and centrifuged, they were inoculated into 9-day-old specific-pathogen-free (SPF) chicken embryos, and allantoic fluid was harvested from the embryos after 72 h of culture. An HA test was used to determine the HA activity. The influenza virus and the HA subtype of the virus were further identified using the hemagglutination inhibition (HI) test with mono-factor serum. The results of the HA activity and HI tests were verified by reverse-transcription PCR (RT-PCR) using subtype-specific primers. The NA subtypes were directly analysed by subtype-specific RT-PCR and sequencing analysis [9].

### Genetic and phylogenetic analyses

Viral RNA was extracted from infected allantoic fluid (TianGen kit, China) and reverse-transcribed (Promega kit, America). We synthesized cDNA using the Unit 12 primer; then, we amplified the eight segments of the influenza virus (sequences available upon request) [10]. The PCR products were purified using a PCR purification kit (TianGen kit, China). Sequencing was performed using the BigDye Terminator v3.1 Cycle Sequencing Kit with an ABI PRISM 3500xl DNA Analyzer (Applied Biosystems). The nucleotide sequences were edited with the Seqman module of the DNASTAR package. Phylogenetic analysis was performed using the MEGA 6.0 software package with the maximum-likelihood method, and the tree topology was evaluated by 1000 bootstrap analyses [11].

#### Pathogenic virus in mice

Groups of 6-week-old female BALB/c mice (Beijing Vital River Laboratories, Beijing, China) were anesthetized with CO_2_ and inoculated intranasally (i.n.) with 10^6^ EID_50_ of test viruses in a volume of 50 μl [12]. Three mice were euthanized on day three post-inoculation (p.i.), and the nasal turbinates, lungs, kidneys, spleens, and brains were collected for virus titration in 10-day-old embryonated chicken eggs. The remaining five mice in each group were monitored daily for 14 days for weight loss and survival [13].

## Results

### Influenza virus isolation and subtype identification

In total, seven H1-subtype AIVs were isolated from 4 534 wild bird faecal samples collected from Anhui Province, China, from 2014 to 2015. The 7 H1 AIVs were all isolated in 2014, including the H1N1 and H1N2 subtypes. Eight genes of the influenza virus were sequenced (the short name of the gene and the gene length are given in parentheses after the descriptive name of the gene): polymerase PB2 (PB2, 2280 bp), polymerase PB1 (PB1, 2274 bp), polymerase PA (PA, 2151 bp), HA (1701 bp) in length, nucleoprotein (NP, 1497 bp), NA (1410 bp), matrix protein 1 (M, 982 bp), and non-structural protein 1 (NS, 844 bp) (GenBank accession nos. KU881669-KU881724). According to species identification before sampling and faecal morphology, the collected viruses were all obtained from birds of the order Anseriformes.

### Phylogenetic analysis of entire genes

**Fig. 1:**
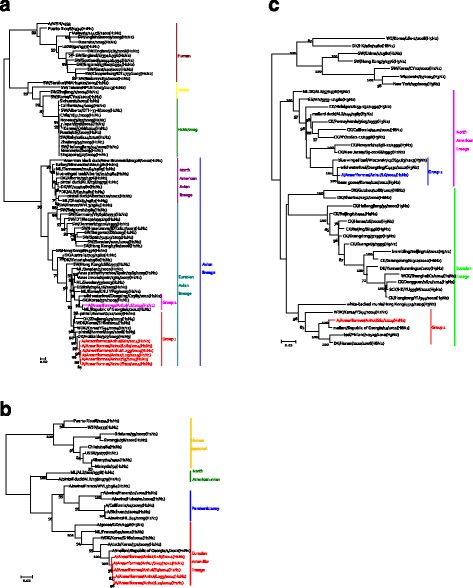
Phylogenetic analysis of the surface genes of H1 subtype AIVs isolated from 2014–2015 by maximum likelihood. The phylogenetic trees were generated with the MAGE 6.0 software package. The phylogenetic trees of the HA (**a**), N1(**b**) and N2(**c**) genes used no root tree. The sequences of viruses listed in *black* were downloaded from available databases; viruses listed in red, *yellow*, and *green* were sequenced in this study. Those listed in cyan and italicized were obtained from the paper by Wang GJ. Abbreviations: CK, chicken; DK, duck; GS, goose; SW, swine; WDK, wild duck; EN, environment

To investigate the phylogenetic relationships of the seven viruses, we sequenced their entire genomes. With the exception of the PA and NS genes of AH/L6 (H1N2), all eight gene segments of the viruses belonged to the Eurasian lineage and displayed distinct diversity. The HA genes of all seven H1 isolates, including five H1N1 viruses and two H1N2 viruses, could be further divided into two groups at the nucleotide level (Fig. 1a). Group 1 contained six H1 AIV strains, and the homology of the strains was > 99.1%. Based on a BLAST search of the GenBank database, all sequences had the highest identity with A/duck/Hokkaido/327/2009 (H1N3). The A/Anseriformes/Anhui/L6/2014(H1N2) virus was in a group by itself; it had the highest sequence identity with A/Mallard/Republic of Georgia/4/2012 (H1N1) (Table 1). N1 genes were found in five strains that belonged to one group in the Eurasian lineage and shared rather high identity (99.0–99.9%); these strains had the highest identity with A/mallard/Republic of Georgia/4/2012 (H1N1). N2 genes were found in two strains that shared 87.9% identity and were divided into two groups. The N2 gene of AH/L6 (H1N2) belongs to the North American lineage, which had the highest identity with A/bean goose/Korea/220/2011 (H9N2); the other N2 gene of AH/S61 (H1N2) belongs to the Eurasian lineage, which had the highest identity with A/mallard/Republic of Georgia/13/201 (H6N2) (Fig. 1b,c).

**Table 1:** The viruses with the highest similarity in GenBank

Virus stains	The highest similarity (%)							
	PB2	PB1	PA	HA	NP	NA	M	NS
A/Anseriformes/Anhui/S3/2014(H1N1)	A/duck/Vietnam/LBM798/2014(H3N6)	A/wildbird/Jiangxi/34458/2013(H7N7)	A/mallard/Republic of Georgia/13/2011(H6N2)	A/duck/Hokkaido/327/2009 (H1N3)	A/duck/Taiwan/11213/2013(H5N2)	A/mallard/Republic of Georgia/4/2012 (H1N1)	A/duck/Vietnam/LBM798/2014(H3N6)	A/duck/Vietnam/LBM533/2013(H3N6)
A/Anseriformes/Anhui/S61/2014(H1N2)	A/common teal/Mongolia/1920/2011(H4N6)	A/duck/Mongolia/200/2015(H3N8	A/Armenian gull/Republic of Georgia/2/2012(H13N2)	A/duck/Hokkaido/327/2009 (H1N4)	A/duck/Quang Ninh/14/2013(H3N6)	A/mallard/Republic of Georgia/13/201 (H6N2)	A/duck/Jiangxi/32213/2013(H7N6)	A/duck/Vietnam/LBM533/2013(H3N6)
A/Anseriformes/Anhui/S107/2014(H1N1)	A/duck/Vietnam/LBM798/2014(H3N6)	A/wildbird/Jiangxi/34458/2013(H7N7)	A/mallard/Republic of Georgia/13/2011(H6N2)	A/duck/Hokkaido/327/2009 (H1N5)	A/duck/Taiwan/11213/2013(H5N2)	A/mallard/Republic of Georgia/4/2012 (H1N1)	A/duck/Vietnam/LBM798/2014(H3N6)	A/duck/Vietnam/LBM533/2013(H3N6)
A/Anseriformes/Anhui/L6/2014(H1N2)	A/bean goose/Korea/220/2011(H9N2)	A/beangoose/Korea/220/2011(H9N2)	A/beangoose/Korea/220/2011(H9N2)	A/Mallard/Republic of Georgia/4/2012 (H1N1)	A/egret/Hunan/1/12(H9N2)	A/beangoose/Korea/220/2011 (H9N2)	A/wildwaterfowl/Dongting/C2150/2011(H9N2)	A/beangoose/Korea/220/2011(H9N2)
A/Anseriformes/Anhui/L25/2014(H1N1)	A/duck/Vietnam/LBM798/2014(H3N6)	A/wildbird/Jiangxi/34458/2013(H7N7)	A/mallard/Republic of Georgia/13/2011(H6N2)	A/duck/Hokkaido/327/2009 (H1N7)	A/egret/Hunan/1/12(H9N2)	A/mallard/Republic of Georgia/4/2012 (H1N1)	A/duck/Vietnam/LBM798/2014(H3N6)	A/duck/Vietnam/LBM533/2013(H3N6)
A/Anseriformes/Anhui/L167/2014(H1N1)	A/ruddy/Mongolia/590c2/2009(H11N2)	A/wildbird/Wuhan/CDHN09/2015(H6N2)	A/commonteal/Mongolia/1920/2011(H4N6)	A/duck/Hokkaido/327/2009 (H1N8)	A/duck/Japan/11OG1032/2011(H5N2)	A/mallard/Republic of Georgia/4/2012 (H1N2)	A/wild waterfowl/Hong Kong/MPP1311/13(H2N9)	A/duck/Vietnam/LBM798/2014(H3N6)
A/Anseriformes/Anhui/L259/2014(H1N1)	A/duck/Vietnam/LBM798/2014(H3N6)	A/wildbird/Jiangxi/34458/2013(H7N7)	A/mallard/Republic of Georgia/13/2011(H6N2)	A/duck/Hokkaido/327/2009 (H1N9)	A/egret/Hunan/1/12(H9N2)	A/mallard/Republic of Georgia/4/2012 (H1N3)	A/duck/Vietnam/LBM798/2014(H3N6)	A/duck/Vietnam/LBM533/2013(H3N6)

**Fig. 2:**
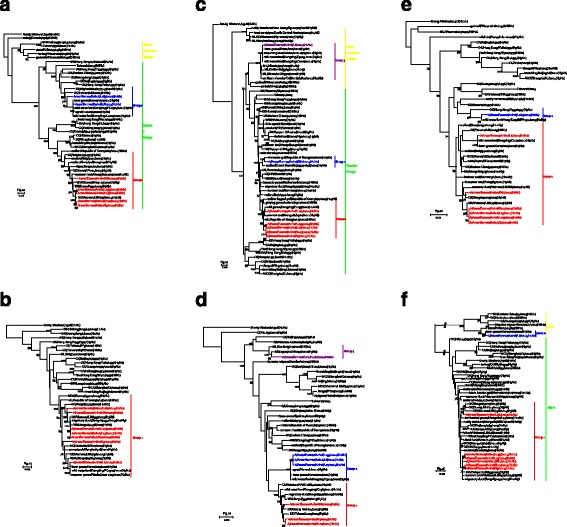
Phylogenetic analysis of the inner genes of H1 subtype AIVs isolated from 2014–2015 by maximum likelihood. The colours of the virus names in the PB2(**a**), PB1(**b**), PA(**c**), NP(**d**), M(**e**), and NS(2f) trees match those used in the genotype table. The phylogenetic trees of inner genes were rooted to A/Spanish/1/1918 (H1N1). The sequences of viruses listed in *black* were downloaded from GenBank; viruses listed in *red*, *yellow*, and *green* were sequenced in this study. Abbreviations: CK, chicken; DK, duck; GS, goose; SW, swine; WDK, wild duck; EN, environment

The PB2 genes were divided into two groups. The nucleotide and amino acid homologies of the seven H1 isolates were 87.5–99.4% and 97.1–99.9%, respectively (Fig. 2a). Seven strains contained PB1 and were clustered into one group; their nucleotide and amino acid homologies were 95.4–100% and 98.8–99.9%, respectively (Fig. 2b). The PA genes were divided into three groups and comprised a mix of the Eurasian branch and the North American branch; their nucleotide and amino acid homologies were 90.2–99.9% and 97.5–99.9%, respectively (Fig. 2c). The NP, M, and NS genes were all divided into two groups (Fig. 2d,e). The NS phylogenetic tree showed that the NS genes of the H6 AIVs were clearly divided into two genetic lineages, termed allele A and allele B; the nucleotide and amino acid homologies of the NS genes were 70.6–100% and 63.2–100%, respectively (Fig. 2f). Each of the virus’s inner genes was blasted with the sequences in GenBank (Table 1).

**Fig. 3:**
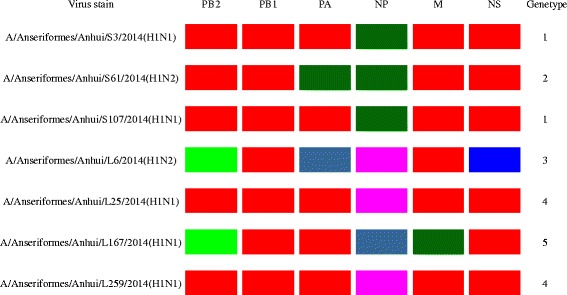
Map of the genotypic evolution of H1Nx viruses. The eight gene segments are indicated at the top of each bar

**Fig. 4:**
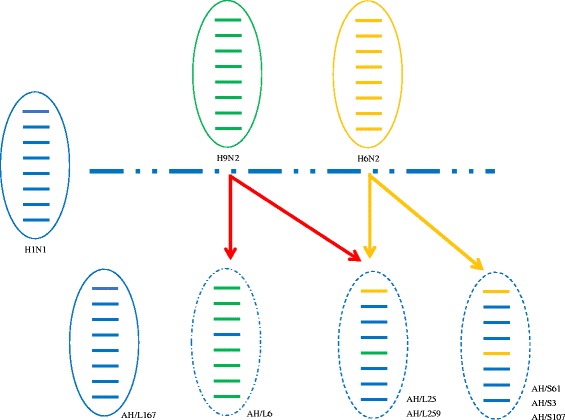
Simplified schematic showing the putative genomic composition of the novel reassortant H1N2 and H1N1 avian influenza viruses described in this study. The eight gene segments (from *top* to *bottom*) in each virus are PB2, PB1, PA, HA, NP, NA, M, and NS. Each colour represents a separate source background. This illustration is based on the nucleotide distance comparison and phylogenetic analysis

The genes encoding internal proteins exhibited more diversity than the genes encoding surface proteins. The seven viruses formed five genotypes according to their genetic diversity (>95%) (Fig. 3). The AH/S3(H1N1) and AH/S107(H1N1) viruses of genotype 1 were a reassortant containing the PB2 and NP genes from the A/Muscovy duck/Vietnam/LBM455/2013(H6N2)-like virus and other genes from the A/duck/Fujian/JF47/2014(H1N1)-like virus. The AH/S61 (H1N2) virus of genotype 2 was a triple reassortant containing the PB2 and NP genes from the A/Muscovy duck/Vietnam/LBM455/2013(H6N2)-like virus, the PA gene from the A/wild bird/Wuhan/WHHN16/2014(H1N1)-like virus, and other genes from the A/duck/Fujian/JF47/2014(H1N1)-like virus. The AH/L6(H1N2) virus of genotype three was a reassortant containing the HA gene from the A/mallard/Republic of Georgia/4/2012(H1N1)-like virus; its other seven genes were derived from the A/bean goose/Korea/220/2011(H9N2)-like virus. The two viruses of AH/L25 (H1N1) and AH/L259 (H1N1) of genotype four were reassortants containing the PB2 gene from the A/Muscovy duck/Vietnam/LBM455/2013(H6N2)-like virus, the NP gene from the A/bean goose/Korea/220/2011(H9N2)-like virus and six genes from the A/duck/Fujian/JF47/2014(H1N1)-like virus. The virus of genotype five was a triple-reassortant containing the PB2 and HA genes from the A/wild bird/Wuhan/WHHN16/2014(H1N1)-like virus, the PA gene from the A/mallard/Republic of Georgia/4/2012(H1N1)-like virus and five genes from the A/duck/Fujian/JF47/2014(H1N1)-like virus (Fig. 4). H1N1 and H1N2 in this study were genetically different from one another.

### Molecular characterization of eight AIV segments

With the exception of AH/S61 (H1N2), the H1 viruses examined in this study all had seven potential glycosylation sites on HA (positions 13, 14, 26, 90, and 290 in HA1 and positions 484 and 583 in HA2). AH/S61 contained six potential glycosylation sites and lacked a site at positions 90 – 92. All of the viruses contained the PSIQSR/G motif at the cleavage site between HA1 and HA2. None of the strains had consecutive basic amino acids in the motif, and all conformed to the characteristics of low-pathogenicity AIVs. Accurate prediction of antigenic changes is important for the production of an influenza vaccine for the coming season. The antigenic sites on HA of avian viruses are highly conserved, likely because of the reduced complexity of the avian immune system [14]. Through analysis of the four antigenic sites (Ca, Cb, Sa, and Sb) comprising approximately 50 amino acid residues in the HA, we found that almost all of the residues, with the exception of a few, were conserved [15] and matched the corresponding Anseriformes sequences. In the Sa site, the amino acids at positions 128 – 129, 156 – 160, and 162 – 167 were PN, KKGT/N, and SPKLSKSN, respectively, which are the characteristic amino acids found at these positions in Anseriformes. In our study, only position 159 of the HA protein of AH/L167 demonstrated a change, to T159A. In the Ca region, the amino acids at positions 140 – 145, 169 – 173, 206 – 208, 224 – 225, and 238 – 240 were SYSGAS, TNNKG, SSK, RG, and DQG, respectively; these amino acids were also conserved, and the sequences represent characteristic amino acid sequences at these sites in Anseriformes. However, position 140 of the HA protein of AH/L167 demonstrated a change to S140P. The amino acid P at position 140 in the HA protein is a conserved amino acid in 2009 H1N1 and in Charadriiformes. In all seven H1 AIVs in our study, the amino acid sequence of the Sb site at positions 187 – 198 was TTS/NEQQSLYQNT/A, which coincided with Anseriformes. The Cb region contained only five amino acids, LLTANS at positions 73 – 78. AH/L25 had a mutation at position 75 (T11A). AH/L6 exhibited 1918 H1N1 and 2009 H1N1-like changes that involved substitution of N by S [15] (Additional file 2: Figure S1, Additional file 3: Figure S2, Additional file 4: Figure S3, and Additional file 5: Figure S4).

The HA receptor-binding region of H1 viruses is located at positions 190 and 225 in HA1 (H3 number in the entire manuscript), which carries E190 and G225 and preferentially binds to α2-3 receptors in birds, whereas D190 and G225 favor both α2-3 and α2-6 receptors in pigs, and H1 HA containing D190 and D225 effectively binds to α2-6 receptors in humans [15]. All seven H1 AIVs described in this article had 190D and 225G, indicating that these viruses likely bind to the avian receptor. A necklace deletion in the NA gene confers enhanced lethality of the virus in mice. In this study, the NA genes of the seven H1 AIVs demonstrated no necklace deletions. The H274Y substitution was observed in all of the NA proteins, suggesting that all of these viruses had oseltamivir resistance [16]. Changes in PB2, such as T271A, E627K, and D701N, may contribute to increased virulence and transmission of influenza viruses in mammals. The H1 viruses that we sequenced in this study displayed none of the above changes. The H1 viruses had no Y436H substitutions in the PB1 protein or T515A substitutions in the PA protein, suggesting low pathogenicity to mammalian and avian hosts. No amino acid substitutions were found in the M2 transmembrane domain, suggesting that this virus strain is sensitive to M2 ion channel inhibitors. An N66S substitution was found in the PB1-F2 protein; this substitution is associated with the increased virulence of the 1918 pandemic virus and that of the highly pathogenic AI H5N1 virus that infects mice and ferrets. The H1 strains in this work had asparagine (N) at amino acid position 66 in PB1-F2. No S31N amino acid substitution was found in the M2 protein, indicating that this viral strain is sensitive to amantadine inhibitors. The mutations of N30D and T215A in M1 are signs that the virulence of the influenza viruses in humans is related to their resistance to the antiviral effects of cytokines such as interferon (IFN), and the D92E mutation in the NS1 protein can increase resistance to IFN. No mutations of residue 92 in the NS1 protein were observed in this study (Table 2).

**Table 2:** Molecular analysis of influenza A subtype H1 viruses from wild birds in Anhui Province

Virus stain	PB2			PB1	PB1-F2	PA	HA		NA	M1		M2	NS
	271 T	627E	701 K	436Y	66 N	515 T	190E	225G	63-65 Neck deletion	30 N	215 T	31S	92D
A/*Anseriformes*/Anhui/S3/2014(H1N1)	+	+	+	+	+	+	+	+	-	D	A	+	+
A/*Anseriformes*/Anhui/S61/2014(H1N2)	+	+	+	+	+	+	+	+	-	D	A	+	+
A/*Anseriformes*/Anhui/S107/2014(H1N1)	+	+	+	+	+	+	+	+	-	D	A	+	+
A/*Anseriformes*/Anhui/L6/2014(H1N2)	+	+	+	+	+	+	+	+	-	D	A	+	+
A/*Anseriformes*/Anhui/L25/2014(H1N1)	+	+	+	+	+	+	+	+	-	D	A	+	+
A/*Anseriformes*/Anhui/L167/2014(H1N1)	+	+	+	+	+	+	+	+	-	D	A	+	+
A/*Anseriformes*/Anhui/L259/2014(H1N1)	+	+	+	+	+	+	+	+	-	D	A	+	+

### Replication of H1 viruses in mice

**Fig. 5:**
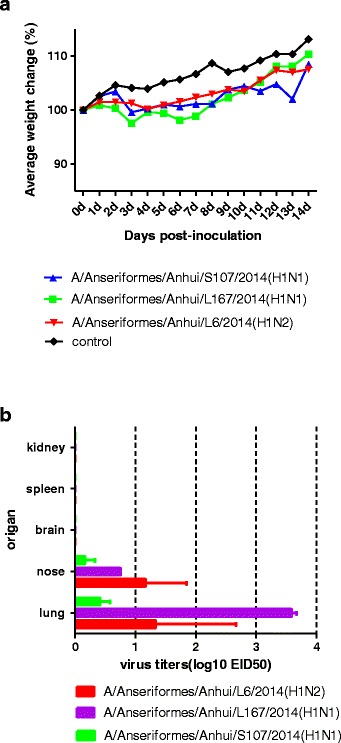
Change in body weight and mortality in BALB/c mice inoculated with H1 viruses. **a** Body weights and survival rates of mice were observed over 14 days after infection. **b** Lungs and tracheas were collected at 3 days post infection (p.i.); the virus replication levels were measured by EID_50_ in specific-pathogen-free (SPF) eggs

BALB/c mice have been widely used as a mammalian model to evaluate the virulence of influenza viruses. To investigate the replication and virulence of these wild bird H1 influenza viruses in mammals, three AIV strains that were deemed representative were tested for replication and lethality in mice. Groups of eight 6-week-old female Balb/c mice were anesthetized with CO_2_ and inoculated i.n. with 10^6^ EID_50_ of each test virus in a volume of 50 μl. Three mice were euthanized on day three p.i., and the nasal turbinates, lungs, kidneys, spleens, brains, eyes, and rectums of the animals were collected for virus titration in eggs. The remaining five mice in each group were monitored daily for 14 days for survival. All three viruses caused the mice to experience body weight loss during the observation period (Fig. 5a). The results of virus proliferation tests in chicken embryos showed that none of the viruses were detected in spleen, kidney, or brain tissue, but all three viruses were detected in the lungs and in the nasal turbinate (Fig. 5b). Virus titers in mice inoculated with AN/L167 were significantly higher than those in mice inoculated with AH/L6 or AH/S107.

To investigate whether any changes occurred in the viral genome during viral replication in mice, we sequenced viruses that had been re-isolated from the infected mice, but we did not identify any mutations. The virus replication levels found in the AH/S107, AH/L6, and AH/L167 infected mice were 0.75 ± 0.14, 0.83 ± 1.44, and 3.58 ± 0.14 log10 EID_50_ in the lungs and 0.17 ± 0.29, 0.08 ± 0.14, and 0.75 log10 EID_50_ in the nasal turbinate, respectively.

## Discussion

The high-level biodiversity of birds (more than 10 000 species) allows AIVs to evolve in different hosts. Birds living near wetlands or aquatic environments, particularly those of the orders Anseriformes (aquatic waterfowls) and Charadriiformes (shorebirds), are considered to provide the gene pool for all influenza viruses [17]. Wild birds are the natural hosts of influenza viruses and can act as a reservoir for the transfer of genes between species, forming transient “genome constellations” that are continually reshuffled by reassortment [17] and providing viral genes during the evolution of new virus species and viruses for interspecies transmission [18]. Furthermore, AIVs are globally distributed due to wild bird migration. This distribution might be underreported due to lack of surveillance and because AIVs can cause mild clinical signs or completely subclinical infections.

Potential pathways for the spread of AIV between countries include international animal trade and wild bird migration. In this study, new reassortment viruses sharing the characteristics of the North American lineage and the Eurasian lineage were identified, including H1N1 and H9N2. The PA and NS genes of AH/L6 (H1N2), which belongs to the North American lineage, had the highest sequence identity with A/bean goose/Korea/220/2011 (H9N2). China and Korea share the East Asian and Australasian migratory bird flyways. Countries located along this flyway share the AIV gene pool [19]. These findings provide strong evidence suggesting not only that direct transmission of viruses occurs between Korea and China via wild birds but also that North American–Eurasian reassortant viruses are presently circulating in wild birds [20], which further indicates that wild birds, particularly migratory birds, play important roles in the reassortment of viruses between two relatively separate and dominant lineages. The seven H1Nx viruses in the present study were novel reassortments carrying genes from H1N1, H6N2, and H9N2 viruses that had been detected previously in ducks and wild birds throughout the world, suggesting that multiple subtypes of the influenza virus may co-circulate in poultry, waterfowl, and migratory birds. Furthermore, the fact that wild birds play important roles in virus reassortment and evolution as natural reservoirs also highlights the affinity between wild birds and poultry from an ecological point of view. To the best of our knowledge, this is the first report of the isolation of H1N1 and H1N2 subtype AIVs in China.

Due to the relatively unsophisticated methods of breeding poultry in China, poultry and wild birds come into frequent contact with one another, making it possible for avian viruses to circulate between wild birds and poultry. This increases the spread of viral mutations and allows new virus strains to be disseminated frequently and easily. Although the H1-subtype influenza viruses of avians and humans show large differences, the potential threat to humans from avian H1 viruses cannot be neglected. We evaluated the potential H1 AIV threat to mammals in this study in a mouse infection experiment. The results showed that H1 AIVs from wild birds can infect mice without adaptation. Therefore, close attention should be paid to all H1N1 virus isolates obtained during future surveillance studies for risk assessment and pandemic preparedness. At the same time, improved feeding management standards will be helpful in the control of AIVs.

Limited information is available, however, to assess exposure levels and risk factors for acquiring avian influenza A virus infections among persons with high levels of contact with wild birds. However, epidemiological studies of human infection with avian influenza A viruses have been conducted among healthcare workers, social contacts, and persons who routinely come into contact with poultry, such as poultry workers, bird cullers, and workers in open bird markets [21, 22]. Studies of persons who have contact with either poultry or wild birds suggest that certain personal protective precautions, such as wearing gloves or eye protection and washing hands after handling birds, should be followed [23–25]. Continued surveillance of AIVs in wild birds is necessary to provide the first line of defence for the prevention and control of AIV infections.

## Conclusions

Seven strains of the H1 subtype avian influenza virus were isolated from migratory and captive birds. Phylogenetic analysis showed that natural recombination of these viruses between the Eurasian lineage and the North American lineage has occurred. These viruses were shown to be able to multiply in mouse respiratory organs. Continued surveillance of wild birds, particularly migratory birds, is important to provide an early warning against AIV outbreaks and to increase our understanding of the ecology of the virus.

## Additional files

Additional file 1: Multilingual abstracts in the five official working languages of the United Nations. (PDF 529 kb)

Additional file 2: Antigenic of Ca analysis of influenza A subtype H1 viruses from wild birds Anhui Province compared with previously isolated H1 viruses. (TIF 79 kb)

Additional file 3: Antigenic sites of Cb analysis of influenza A subtype H1 viruses from wild birds in Anhui Province compared with previously isolated H1 viruses. (TIF 78 kb)

Additional file 4: Antigenic sites of Sa analysis of influenza A subtype H1 viruses from wild birds in Anhui Province compared with previously isolated H1 viruses. (TIF 123 kb)

Additional file 5: Antigenic sites of Sb analysis of influenza A subtype H1 viruses from wild birds in Anhui Province compared with previously isolated H1 viruses. (TIF 84 kb)

## Abbreviations

AIV: Influenza A virus
HA: Hemagglutinin
M: Matrix
NA: Neuraminidase
NP: Nucleoprotein
NS: Non-structural
PA: Acidic polymerase
PB1: Polymerase basic 1
PB2: Polymerase basic 2
RT-PCR: Reverse transcription polymerase chain reaction
