# Supplementary material for: Epidemic of wild-origin H1NX avian influenza viruses in Anhui, China
Source: Infect Dis Poverty. 2017 Jul 3;6:98. doi: 10.1186/s40249-017-0304-4 (PMC5494855; doi:10.1186/s40249-017-0304-4)
Supplement: Supplementary file 1 — Multilingual abstracts in the five official working languages of the United Nations. (PDF 529 kb) [file 40249_2017_304_MOESM1_ESM.pdf]

Translation of the abstract into the five official working languages of the United Nations

وباء فيروسات الأنفلونزا H1NX الناشئة من الطيور البرية في أنهوى، الصين

يي جي، تشيو تشنج ياو، شيان فو وانج، زي تشيانج فان، جوا هوا دنج، هونج ليانج تشاي، هوا لان تشن آند يو بينج هوا

خلاصة

خلفية: باعتبارها الحاضن الطبيعي لفيروسات إنفلونزا الطيور (AIVs)، توفر الطيور المائية والمهاجرة تجمع جيني لنقل الجيني بين الأنواع وضمن الأنواع، مشكلة "تجمعات جينوم" عابرة. يصف هذا العمل ديناميكيات النشوء والتطور لفيروس H1NX بناء على التوصيف الجزيئي الكامل لثمانية جينات للفيروسات التي جمعت في الفترة 2014 و2015 في مقاطعة أنهوى، الصين.

أساليب: استخدمت اختبارات التراص وتثبيط التراص لتحديد نشاط التراص (HA) للأنواع الفرعية للتراص. تم عمل تسلسل للجينومات الكاملة للفيروسات على محلل الحمض النووي ABI PRISM 3500xl. وقد تم تحليل التسلسل وراثيا لدراسة التطور الجيني باستخدام DNASTAR وMEGA 6. تم تقييم الآثار المسببة للأمراض الناشئة من الفيروسات باستخدام نماذج عدوى الفئران.

النتائج: تم عزل سبعة سلالات من فيروس انفلونزا الطيور النوع الفرعي H1. وأشار تحليل النشوء والتطور لإعادة تركيب طبيعي لفيروسات أنفلونزا H1 بين سلالة أوراسيا وسلالة أمريكا الشمالية. وكان لبعض الجينات هوية تسلسل عالية مع A/bean goose/Korea/220/2011 (H9N2)، وهي حالة نموذجية تشمل إعادة الترتيب الفيروسي بين سلالة أوراسيا وسلالة أمريكا الشمالية. وأظهرت نتائج تجارب العدوى في الفئران أن الفيروسات يمكن أن تكتسب القدرة على التكاثر في الجهاز التنفسي في الفأر دون تكيف.

الاستنتاجات: تشير هذه النتائج إلى أن استمرار مراقبة الطيور البرية وخاصة الطيور المهاجرة، هام لتوفير الإنذار المبكر لأوبئة الأنفلونزا H1 الممكن حدوثها وفهم بيئة الفيروس.

Translated from English version into Arabic by Mahmoud Sami, through

TRANSLATORS
WITHOUT BORDERS

安徽省野鸟源 H1NX 亚型禽流感病毒的流行病学分析

葛叶，姚秋成，王先福， 范志强，邓国华，柴洪亮，陈化兰，华育平

摘要

引言：野生水禽和迁徙鸟类作为流感病毒的自然宿主为流感病毒的传播和跨物种传播提供基因池，形成瞬时“基因组集合”。本研究对 2014-2015 年度中国安徽省野鸟中 H1NX 亚型禽流感病毒开展了病毒的分离鉴定、遗传演化和分子特征评价等工作。

方法：通过血凝实验和血凝抑制实验鉴定流感病毒HA亚型。采用ABI PRISM 3500xl测序仪对病毒的全基因组序列测定。利用DNASTAR和MEGA 6.0软件进行基因组的遗传演化分析。通过小鼠的感染模型评价病毒对哺乳动物潜在的致病性。

结果：本研究共分离到7株H1亚型禽流感病毒。遗传演化分析结果表明这7株病毒存在着北美分支病毒和欧亚分支病毒的重组现象。一些基因与毒株A/bean goose/Korea/220/2011(H9N2)的基因高度同源，而该毒株是北美分支病毒和欧亚分支病毒的重组病毒的典型代表。小鼠的感染性实验结果显示，本实验分离到的H1亚型禽流感病毒具有不经适应直接在小鼠的呼吸

器官复制的能力。
结论：本研究结果显示，持续进行野鸟，尤其是迁徙鸟类的禽流感病原学监测工作，为H1亚型流感病毒的流行及病毒的生态进化提供早期的防控预警和第一手资料。

Translated from English version into Chinese by Ye Ge

L'Épidémie des virus de l'influenza aviaire H1NX d'origine sauvage en Anhui, en Chine

Ye Ge, Qiu-Cheng Yao, Xian-Fu Wang, Zhi-Qiang Fan, Guo-Hua Deng, Hong-Liang Chai, Hua-Lan Chen et Yu-Ping Hua

Résumé

Contexte : En tant qu'hôtes naturels des virus de l'influenza aviaire (VIA), les oiseaux migratoires et aquatiques fournissent un bassin génétique pour le transfert génétique entre les espèces et d'une espèce à l'autre, ce qui forme des « constellations de génomes » transitoires. Ce travail décrit les dynamiques phylogénétiques des virus H1NX selon la caractérisation moléculaire complète de huit gènes de virus qui ont été recueillis entre 2014 et 2015 dans la province de l'Anhui, en Chine.
Méthodes : Des tests d'inhibition de l'hémagglutination ont été effectués pour déterminer l'activité de l'hémagglutination (AH) des sous-types d'AH. Les génomes complets des virus font l'objet de séquençage au moyen d'un analyseur d'ADN ABI PRISM 3500xl. Ensuite, l'analyse générique des séquences a été réalisée dans le but d'étudier leur évolution génétique au moyen de DNStar et MEGA 6. Les effets pathogènes des virus ont été évalués à l'aide de modèles d'infection (souris).
Résultats: Sept souches du sous-type H1 des virus de l'influenza aviaire ont été isolées. Des analyses phylogénétiques ont indiqué qu'il y avait une recombinaison naturelle des virus de l'influenza H1 entre la lignée eurasienne et la lignée nord-américaine. Certains gènes ont présenté une identité de séquence élevée avec A/oie des moissons/Corée/220/2011 (H9N2), ce qui représente un cas typique de réassortiment génétique du virus entre la lignée eurasienne et la lignée nord-américaine. Les résultats des expériences d'infection chez des souris ont démontré que les virus pourraient se multiplier dans les organes respiratoires des souris sans adaptation.
Conclusions: Selon ces résultats, la surveillance continue d'oiseaux sauvages, surtout les oiseaux migratoires, est importante afin de fournir une alerte rapide d'épidémies possibles en raison des virus de l'influenza H1 et pour comprendre l'écologie du virus.

Translated from English version into French by Edith Emilie Mercier, through

TRANSLATORS WITHOUT BORDERS

Эпидемия вирусов птичьего гриппа H1NX дикого происхождения в провинции Аньхой, Китай

Е Гэ, Цю-Шен Яо, Сиань-Фу Ван, Чжи-Цян Фан,Го-Хуа Дэн, Хун-Лян Чай, Хуа-Лан Чен и Ю-Пинь Хуа

Реферат статьи

Введение: Водные и перелетные птицы, в качестве естественных хозяев вирусов птичьего гриппа (AIVs), обеспечивают генофонд для их наследственного переноса между видами и от одного вида к другому, образуя переходные "геномные созвездия". Эта работа описывает филогенетическую динамику вируса H1NX на основе полной молекулярной характеристики восьми генов вирусов, что были собраны в провинции Аньхой (КНР) с 2014 по 2015 год.

Методы: Для определения активности гемагглютинации (ГА) подтипов НА проводились исследования на гемагглютинацию и ее торможение. Все геномы вирусов были секвенированы с помощью ДНК анализатора ABI PRISM 3500xl. Анализ последовательностей с целью изучения их генетической эволюции был выполнен с помощью DNAstar и MEGA 6. Патогенные эффекты вирусов оценивались с использованием моделей заражения мышей.

Результаты: были выделены семь штаммов вируса птичьего гриппа подтипа H1. Филогенетический анализ показал естественные рекомбинации вирусов гриппа H1 между евразийской и североамериканской линиями. Некоторые гены имели высокую идентичность последовательности со штаммом A/bean goose/Korea/220/2011(H9N2), который представляет собой типичный случай вирусной рекомбинации между евразийской и североамериканской линиями. Результаты экспериментов с заражением мышей показали, что вирусы могут приобретать способность размножаться в дыхательных органах мышей без адаптации.

Выводы: Полученные данные свидетельствуют о том, что для обеспечения раннего предупреждения о возможных эпидемиях гриппа H1 и понимания механизмов его взаимодействия с человеком, необходим постоянный надзор за дикими птицами (в особенности перелетными).

Translated from English version into Russian by datran, through

Epidemia de virus H1NX de gripe aviar de origen silvestre en Anhui, China

Ye Ge, Qiu-Cheng Yao, Xian-Fu Wang, Zhi-Qiang Fan, Guo-Hua Deng, Hong-Liang Chai, Hua-Lan Chen and Yu-Ping Hua

Resumen

Antecedentes: Al igual que los huéspedes naturales de virus de la gripe aviar (AIVs *por sus siglas en inglés*), las aves acuáticas y migratorias suministran un fondo genético para la transferencia genética mediante especies y por medio de especies, formando "constelaciones de genoma" fugaces.

Este trabajo describe la dinámica filogenética de H1NX basada en la caracterización molecular completa de los ocho genes de virus que se recogieron de 2014 a 2015 en la provincia de Anhui, China.

Métodos: Se utilizaron ensayos de hemoaglutinación e inhibición de la hemoaglutinación para determinar la actividad de hemoaglutinación (HA) de los subtipos HA. Los genomas completos de los virus se secuenciaron sobre un analizador de ADN ABI PRISM 3500XL. Las secuencias se analizaron, genéticamente, para estudiar su evolución genética utilizando DNASTAR y MEGA 6. Los efectos patógenos de los virus se evaluaron utilizando modelos de ratón de infección.

Resultados: Se aislaron siete cepas del virus de la gripe aviar de subtipo H1. El análisis filogenético indicó una recombinación natural de virus de la gripe H1 entre el linaje euroasiático y el linaje norteamericano. Algunos genes tuvieron una alta identidad de secuencia con A / *bean goose*/ Corea / 220/2011 (H9N2), que es un caso típico que implica un intercambio viral entre el linaje euroasiático y el linaje norteamericano. Los resultados de experimentos de infección en ratones mostraron que los virus podían adquirir la capacidad de multiplicarse en los órganos respiratorios de ratón sin adaptación.

Conclusiones: Estos hallazgos sugieren que es importante la continua vigilancia de las aves silvestres, particularmente, las aves migratorias, para advertir, a tiempo, las posibles epidemias de gripe H1 y para comprender la ecología del virus.

Translated from English version into Spanish by Maria Luz Puert, through

TRANSLATORS
WITHOUT BORDERS
