# Supplementary figures and images for: Epidemic of wild-origin H1NX avian influenza viruses in Anhui, China
Source: Infect Dis Poverty. 2017 Jul 3;6:98. doi: 10.1186/s40249-017-0304-4 (PMC5494855; doi:10.1186/s40249-017-0304-4)

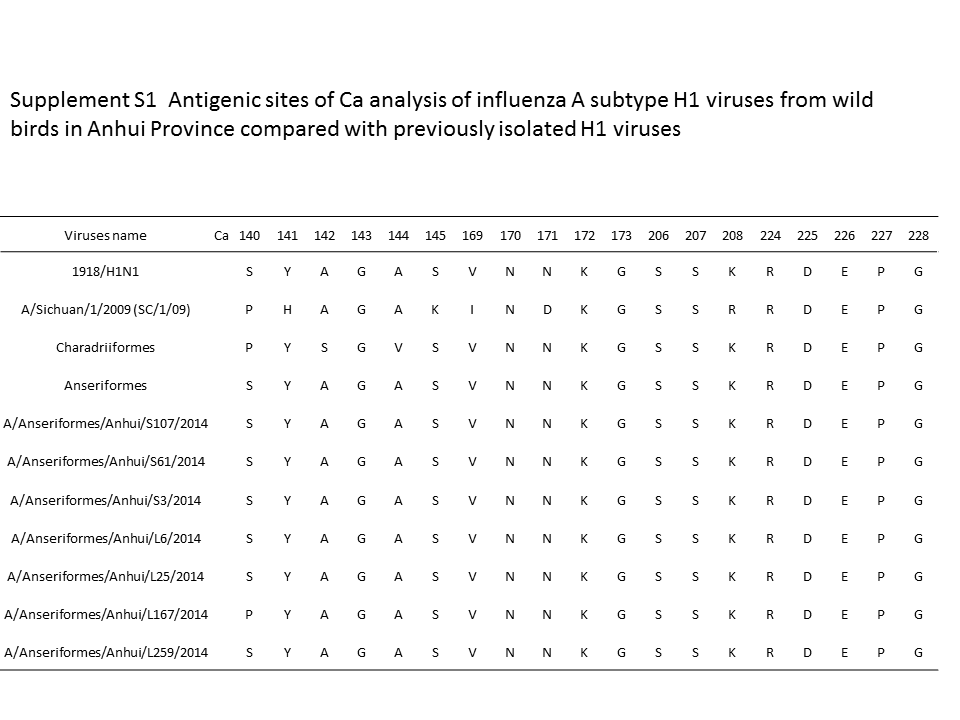

Supplement: Supplementary file 2 — Antigenic of Ca analysis of influenza A subtype H1 viruses from wild birds Anhui Province compared with previously isolated H1 viruses. (TIF 79 kb) [file 40249_2017_304_MOESM2_ESM.tif]

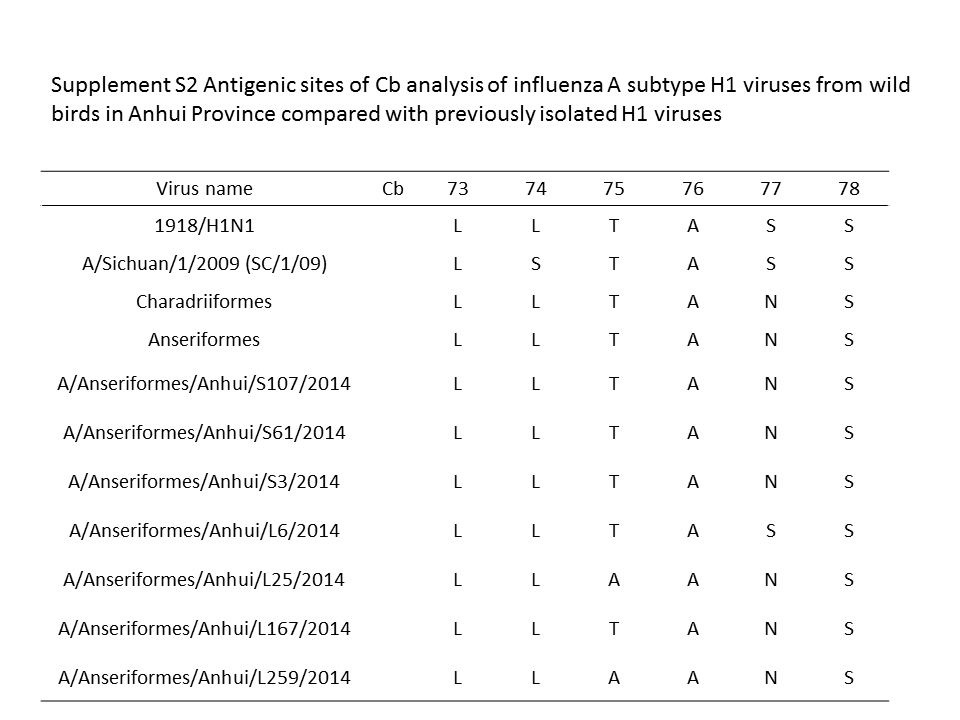

Supplement: Supplementary file 3 — Antigenic sites of Cb analysis of influenza A subtype H1 viruses from wild birds in Anhui Province compared with previously isolated H1 viruses. (TIF 78 kb) [file 40249_2017_304_MOESM3_ESM.tif]

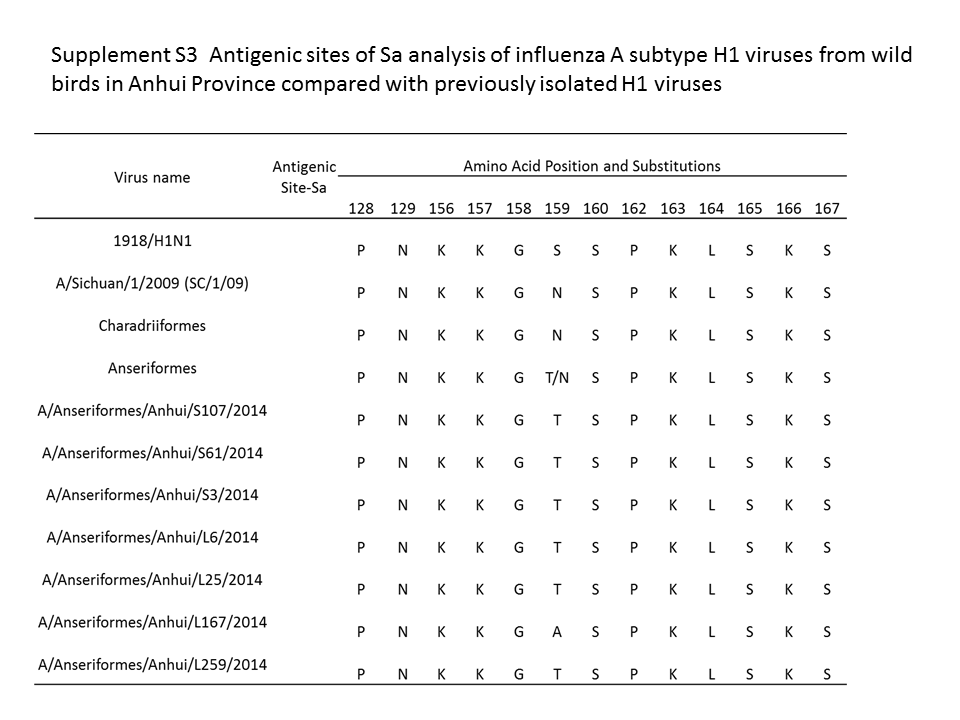

Supplement: Supplementary file 4 — Antigenic sites of Sa analysis of influenza A subtype H1 viruses from wild birds in Anhui Province compared with previously isolated H1 viruses. (TIF 123 kb) [file 40249_2017_304_MOESM4_ESM.tif]

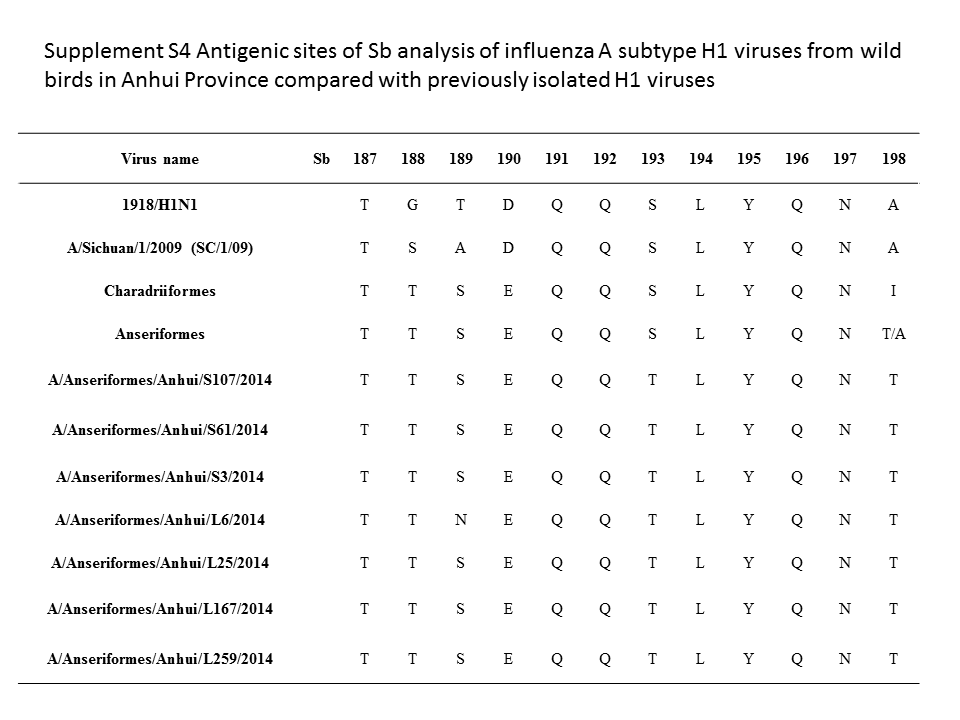

Supplement: Supplementary file 5 — Antigenic sites of Sb analysis of influenza A subtype H1 viruses from wild birds in Anhui Province compared with previously isolated H1 viruses. (TIF 84 kb) [file 40249_2017_304_MOESM5_ESM.tif]
